# Change of Accent as an Atypical Onset of non Fluent Primary Progressive Aphasia

**DOI:** 10.3233/BEN-120299

**Published:** 2013

**Authors:** Susy Paolini, Lucia Paciaroni, Antonio Manca, Roberto Rossi, Daniela Fornarelli, Stefano F. Cappa, Angela M. Abbatecola, Osvaldo Scarpino

**Affiliations:** ^1^Unit of NeurologyItalian National Research Center on AgingVia della MontagnolaAnconaItaly; ^2^Unit of RadiologyItalian National Research Center on AgingAnconaItaly; ^3^Vita-Salute University and Division of NeuroscienceSan Raffaele Scientific InstituteMilanItaly; ^4^Scientific DirectionItalian National Research Center on AgingAnconaItaly

**Keywords:** Dementia, primary progressive aphasia, progressive non fluent aphasia, dysprosodic disorder, foreign accent syndrome

## Abstract

Language disorders can be the first symptom of many neurodegenerative diseases, including Alzheimer's disease (AD) and primary progressive aphasia (PPA). The main variants of PPA are: the non-fluent/agrammatic variant, the semantic variant and the logopenic variant.

Several additional variants of PPA, however, have been described and are considered as atypical presentations.

We describe the case of a woman presenting a progressive isolated language disturbance, characterized by an early dysprosodia, phonological and semantic paraphasias, agrammatism, impairment in repetition, writing of non-words and sentence comprehension. This clinical picture pointed to an atypical presentation of the non-fluent variety. The frequent symptom overlap between the different variants of PPA, most likely reflecting differences in the topography of the pathological changes, needs to be considered in the definition of diagnostic criteria.

## 1. Introduction

Isolated language disturbances can be the first symptoms of fronto-temporal lobar degeneration (FTLD). In 1982, Mesulam first introduced Primary Progressive Aphasia (PPA) [1,2] to describe an isolated language impairment that manifests in an insidious manner, remains isolated for at least two years and then evolves into dementia. Subsequent studies identified different clinical presentations of PPA and at the present, three main variants of PPA have been described as: progressive non fluent aphasia, semantic dementia, and logopenic progressive aphasia [3–5].

In 2011, the International Consensus Criteria [6] adopted the following three clinical subtypes for the classification of PPA: nonfluent/agrammatic variant PPA (PPA-NFV), semantic variant PPA (PPA-SV) and logopenic variant PPA (PPA-LV) (Table 1). PPA-NFV is characterized by an effortfull and halting speech with agrammatism, possible anomias and phonologic paraphasias [3,5,7]. Patient comprehension is preserved for single words, while it is slightly impaired for sentences, especially for difficult morphosyntactic constructions [8]. In this variant, apraxia of speech, dysarthria, stuttering, impaired repetition, alexia and agraphia can be found without severe amnesia and/or perceptuo-spatial disorder [5]. Studies of structural and functional imaging suggest an involvement of left inferior frontal region and left anterior insular cortex [7, 9,10]. Clinical presentation of the PPA-SV is associ-

ISSN 0953-4180/13/$27.50 © 2013 – IOS Press and the authors. All rights reserved

Table 1
Addendum criteria for subtypes of PPA [6]

| |
|---|
| Clinical diagnosis of nfvPPA |
| At least one of the following core features must be present: |
| 1- Agrammatism in language production |
| 2- Effortful, halting speech with inconsistent speech sound errors and distortions (apraxia of speech) |
| At least 2 of the 3 following other features must be present: |
| 1- Impaired comprehension of syntactically complex sentences |
| 2- Spared single-word comprehension |
| 3- Spared object knowledge |
| Clinical diagnosis for svPPA |
| Both of the following core features must be present: |
| 1- Impaired confrontation naming |
| 2- Impaired single-word comprehension |
| At least 3 of the following other diagnostic features must be present: |
| 1- Impaired object knowledge, particularly for low-frequency or low-familiarity items |
| 2- Surface dyslexia or dysgraphia |
| 3- Spared repetition |
| 4- Spared speech production (grammar and motor speech) |
| Clinical diagnosis for lvPPA |
| Both of the following core features must be present: |
| 1- Impaired single-word retrieval in spontaneous speech and naming |
| 2- Impaired repetition of sentences and phrases |
| At least 3 of the following other features must be present: |
| 1- Speech (phonologic) errors in spontaneous speech and naming |
| 2- Spared single-word comprehension and object knowledge |
| 3- Spared motor speech |
| 4- Absence of frank agrammatism |

ated with normal fluency, as well as an impairment of object naming, deficit of single words comprehension and surface dyslexia, all explained by the disruption of semantic knowledge [8,11–14]. Generally, patients with PPA-SV have a bilateral atrophy of anterior and inferior temporal lobes that is more extensive in the left hemisphere [15–17]. Finally, PPA-LV patients exhibit word finding difficulties and decreased output, impaired naming and repetition in the context of spared semantic and syntactic abilities, while mantaining syntactically simple correct language output [3, 18]. Phonemic paraphasias are also frequent, as well as an impairment in sentence comprehension especially for long sentences, whereas single word comprehension and semantic memory are preserved [18,19]. In these patients, atrophy is localized in the posterior temporal and inferior parietal regions of dominant hemisphere [16,18,19].

In the literature, diverse clinical presentations of language progressive disorders that do not fit the recent criteria have been described, including progressive anarthria [20–23] and progressive jargon aphasia [24]. Some authors have hypothesized that some of these clinical variants could represent different stages of the same disease or an atypical presentation due to variations in specific areas of cerebral degeneration [25].

Regarding the PPA-NFV, a recent study by Luzzi et al. [26] reported an atypical onset of the foreign accent syndrome (FAS). These authors reported a case of an Italian woman presenting a progressive change in her accent, so that listeners perceived her as a foreigner. No other linguistic or cognitive disorders were observed at onset and after one year, a PPA-NFV was diagnosed.

In this report, we also describe an atypical onset of PPA-NFV in a woman presenting as first symptom a prosodic change perceived as a regional accent change rather than as a foreign accent. The change of regional accent has been already described in different diseases and is considered as a variant of FAS [27,28]. We suggest that it may represent an atypical feature of PPA-NFV presentation.

## 2. Case report

### 2.1. Clinical details

A 78-year right-handed Italian woman with 8 years of formal education came to our attention in March 2008 for a language disorder characterized by dysprosodia, with sporadic phonologic and semantic paraphasias that had been evolving over the last two years.

Her family members claimed that her symptoms began with a progressive change in the loudness and pitch

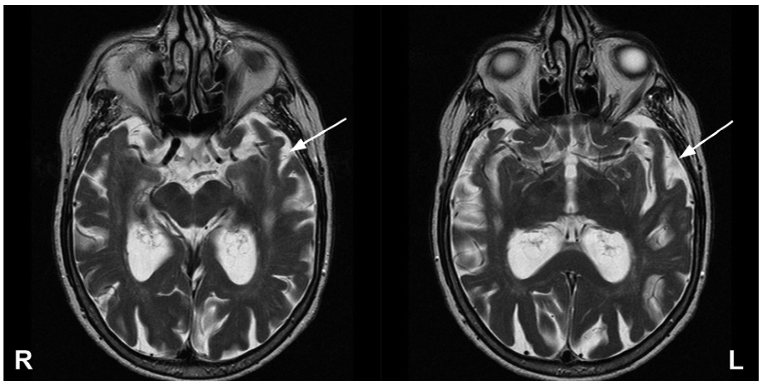

Fig. 1. MRI-scan: Spin Echo sequences T2-weighted, parallel to the long axis of the temporal lobe, showed cortical atrophy more evident in the left side.

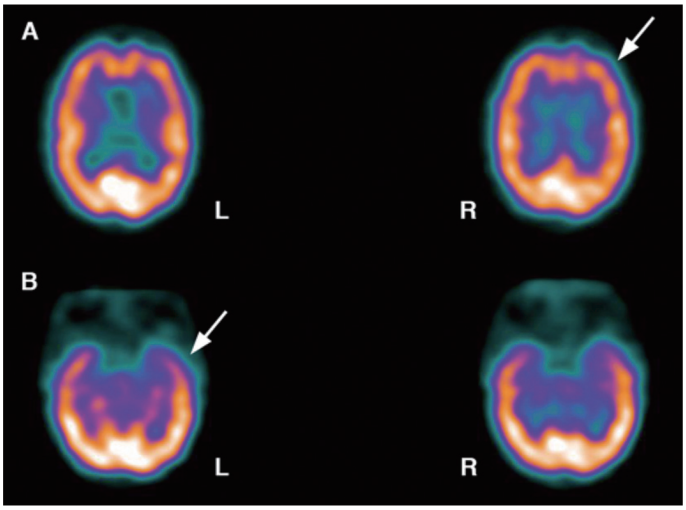

Fig. 2. 99m-Tc-ECD-SPECT evidenced hypoperfusion in the lateral frontal regions (A) and in the surface and mesial temporal lobes, especially in the left side (B).

of the voice. Her speech was perceived as having undergone a regional accent change.

She lived alone and she was entirely independent in activities of daily living. Minimal behavioural changes were also reported (disinhibition, impulsiveness, euphoria and irritability). Her past medical or psychiatric history was unremarkable and her neurological examination was normal.

### 2.2. *Neuroimaging*

During the first clinical examination, she underwent a MRI brain scan showing cortical atrophy, especially in the left temporal hemisphere (Fig. 1). A 99m-Tc-ECD-SPECT was also performed and evidenced hypoperfusion of the lateral frontal regions especially in the left hemisphere, as well as in lateral and medial temporal lobes (Fig. 2).

### 2.3. *Neuropsychological evaluation*

A comprehensive neuropsychological testing battery, including attention, executive functions, memory, praxis and visuo-spatial abilities was performed, and the results are shown in Table 2.

A mild frontal executive dysfunction was evident, while there were no deficits in visual and verbal episodic memory, visuo-spatial abilities and object/people knowledge. An impairment in short-term memory was found with a low performance in the Digit Span test. She also showed a mild bucco-linguo-facial apraxia.

A detailed language evaluation showed agrammatic, dysprosodic, anomic spontaneous speech with phono-

Table 2
Neuropsychological testing (scores adjusted for age and education according to published norms)

| Tests | Baseline | Follow-up (12 month) | Normal values |
|---|---|---|---|
| Mini mental state examination | 26 | 9.7 | > 24 |
| *Attention and executive functioning* | | | |
| Attentional matrices | 47.5 | 43 | > 31 |
| Trail making test | | | |
| Part A | 29 | 40 | < 93 |
| Part B | 232 | | < 282 |
| Weigl's sorting test | 7.25 | 4.5 | > 4.25 |
| FAB | 5.9* | 4.2* | > 13.4 |
| Stroop test (Time) | 22.25 | | < 36.92 |
| *Memory* | | | |
| Corsi test | 5.25 | | > 3.5 |
| Digit span | 3.25* | 2.5* | > 3.5 |
| Rey AVLT (Immediate recall) | 35.9 | | > 28.52 |
| Rey AVLT (Delayed recall) | 9.6 | | > 4.68 |
| *Visuospatial and constructional skill* | | | |
| Rey complex figure copy | 30 | 28.7 | > 23.76 |
| Praxis | | | |
| Ideomotor praxis | 20 | | > 16 |
| Bucco-linguo-facial praxis | 12,25* | | > 16 |
| *Language* | | | |
| Noun naming (ENPA) | 8/10* | 1/10* | > 8.2 |
| Verb naming (ENPA) | 6.5/10 | 5.5/10* | > 6.1 |
| Token test | 18.25* | 10.75* | > 26.25 |
| Oral words comprehension (ENPA) | 20/20 | 15.6/20* | > 18.4 |
| Animal fluency | 12.5 | 8* | > 9 |
| Phonemic fluency | 1.9* | 0* | > 5.8 |
| Pyramids and palms test | 45,78 | | > 40.15 |

*Pathological score. Abbreviations: FAB = Frontal Assessment Battery; Rey AVLT = Rey Auditory Verbal Learning Test; ENPA = Esame Neuropsicologico per l'Afasia (Capasso R., Miceli G. Esame Neuropsicologico per l'Afasia. Milano: Springer; 2001).

logic errors and rare semantic paraphasias. Although she was born in Marche region (located in the central Italy), where she permanently lived, she gradually began to present a change in her native accent over the last two years. Her accent change was evaluated by 6 native speaking Italians that listened to her speech and all of them judged her accent as similiar to that of the Veneto region (located in the north-east of Italy).

In addition, her prosodic comprehension was impaired for both linguistic (question, command, statement) and affective prosody (angry, sad, surprised, happy, sarcastic). For example, she was unable to distinguish if pairs of sentences were identical or if they differed in terms of intonation or location of stress. During the examination she was not troubled and was unaware of her dysprosody.

Results of formal language testing are reported in Fig. 3 and Table 2. The production tasks showed a mild to moderate naming impairment (more severe for verbs compared to nouns), low phonemic fluency and normal semantic fluency. Single word comprehension was good for nouns and slightly impaired for verbs. Sentence comprehension was severely affected.

Repetition and writing of non-words were seriously impaired, whereas a mild deficit was found for words. Her reading ability was good for both words and non-words.

A brief neuropsychological evaluation was performed at the 12-month of follow up and results underlined a severe worsening on oral production and comprehension, with a relative preservation of time and space orientation, memory in daily living, visuospatial abilities and selective attention. A mild executive deficit was also confirmed.

At the 24-month of follow-up she was almost mutacic and oral comprehension was severely impaired, but was able to read and to recognize familiar faces. Her family members confirmed an overall worsening of her behaviour with impulsivity, aggression and disinhibition.

## 3. Discussion

Neurodegenerative diseases that manifest with language disorders include an overlapping of diverse neu-

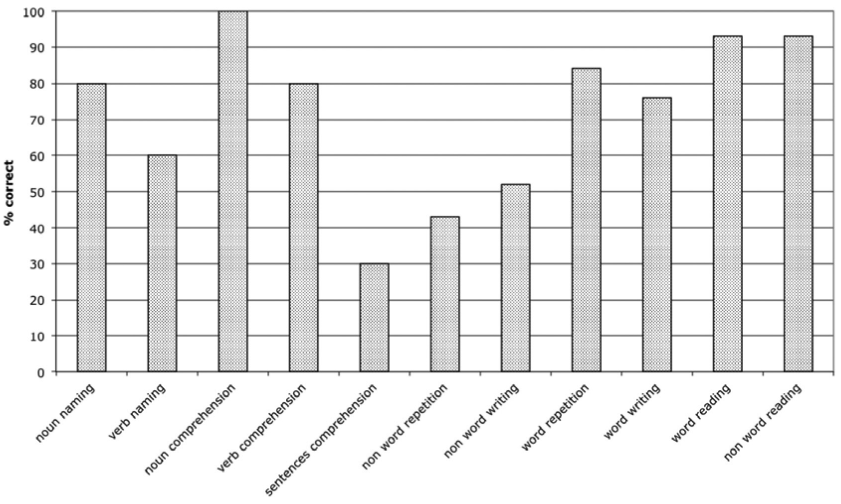

Fig. 3. Performances on BADA Aphasia Examination. BADA = Batteria per l'Analisi del Deficit Afasico (Miceli G. et al. B.A.D.A. Batteria per l'Analisi dei Deficit Afasici. Roma: CEPSAG; 1994).

ropsychological patterns, which may be problematic for a differential diagnosis between an atypical form of Alzheimer's disease (AD), PPA-NFV, PPA-SV and PPA-LV. Indeed, a correct differential diagnosis is extremely important because different clinical patterns may be correlated to distinct neuropathological substrates [29]. In general PPA-NFV is highly associated with tauopathies [24,30,31], while PPA-SV is often due to progranulin/TDP43 pathologies [32–34] and PPA-LV to AD pathology [18,31,33].

Our patient presented with a prominent language disorder, accompanied by an integrity of episodic memory, attention and visuo-spatial abilities. The clinical history of the patient, as reported by her relatives, confirmed an isolated language impairment that had been progressing over the last two years, thus fulfilling the criteria for PPA.

The patient presented frequent anomias and sporadic semantic paraphasias. This could raise the suspicion of the PPA-SV form, given also the cortical atrophy in temporal lobes shown by MRI. However, she had excellent single word comprehension, preserved semantic knowledge and agrammatism, which are incompatible with PPA-SV. The patient also showed moderate difficulties in naming (Table 2; Fig. 3). A qualitative analysis of the errors was performed, and showed that different types of errors were made in response to object and action pictures. Regarding object nouns, the patient presented prevalently phonological errors, whereas for action pictures (verbs) we observed predominantly anomias, circonlocutions and semantic paraphasias, substituting nouns for the target verbs. This finding is atypical for PPA-SV [35]. Furthermore, during her first clinical evaluation, we found difficulties in repetition, which is typically intact in early PPA-SV. Despite the MRI findings of temporal atrophy, this linguistic pattern excluded a PPA-SV.

Patients with PPA-LV usually show a pattern of speech output that is slow, syntactically simple but correct, with frequent word-finding pauses, naming difficulties, phonemic paraphasias and impaired repetition [18,19]. In this variant, the core problem is a deficit in phonological short-term memory [3,19,36], which is related to involvement of the inferior parietal lobule [36–38]. Our patient presented a phonological short-term memory impairment. Furthermore, her difficulty in single word retrieval, in repetition and the speech phonological errors were similar to PPA-LV. Nevertheless, the presence of agrammatism in production, the presence of dysprosodia/regional accent syndrome and the bucco-faccial apraxia are atypical for this variant.

The patient shared many of the diagnostic features of PPA-NFV [6], such as agrammatism in language production, impaired comprehension of syntactically complex sentences, preservation of single word comprehension and object knowledge. Over time her clinical symptoms evolved toward mutism, as expected in PPA-NFV.

Her dysprosodic disorder was an additional clinical sign for PPA-NFV. Disrupted prosody is often found in advanced PPA-NFV as the consequence of articulation disturbances. Only one case of an Italian patient showing dysprosody as early onset of PPA-NFV has been reported [26]. Three years before the onset of PPA-NFV, this patient presented a speech disorder characterized by the foreign accent syndrome (FAS), that is a linguistic prosody disorder in which a new accent is perceived by listeners as foreign. This prosodic disorder is distinct from apraxia of speech, dysathria and aphasic output disorder, but it can occur at the same time [39]. Generally, FAS emerges as consequence of damage to the language dominant frontal systems underlying speech. The main areas affected are the primary motor cortex, cortico-cortical connections and cortico-subcortical projections [39–41]. FAS is also distinct from emotional or affective dysprosody, which has been conceptualised as a dominant and lateralized function of the right hemisphere [40]. Thus, both hemispheres are necessary for successful prosodic performances.

In comparison to the case reported by Luzzi et al. [26], our patient was perceived as a native Italian, but with a significant change in her regional accent. It is often acknowledged that regional and foreign accents share the same mechanisms in production and comprehension. A recent report [42] suggests a continuum between regional and foreign conditions. The damage within the motor speech network can give rise to specific phonological changes, resulting in a different accent, including ones that sound like a regional change, rather than a FAS [42]. Both regional and foreign accents show the same pattern in comprehensibility and intellegibility of the listener [43]. Furthermore, the close relationship between the two conditions is supported by the linguistic theory, confirming that a dialect is a language, as reported in the statement by Max Weinreich [44]: “A language is a dialect with an army and a navy”. For these reasons, our patient may be considered as having a variant of FAS.

In addition, she showed a more severe aphasic picture than the case reported by Luzzi [26], probably because she came to our observation later. She also presented an affective dysprosodia, either in production or in comprehension. Thus, her dysprosodic disorder was characterized by both a linguistic and affective dysprosody, probably because the atrophy interested not only the left linguistic areas, but also the corresponding right ones.

We suggest that the pattern of dysprosodic disorder as the onset of PPA-NFV can vary in function of the prevalent atrophy. If the damage is more evident in the left side, the dysprosodic disorder is characterised by linguistic dysprosodia as in FAS. If atrophy is bilateral then a disorder of affective aspects of prosody will also be associated with linguistic dysprosodia.

Taken together the linguistic findings of our patient were consistent with a probable diagnosis of PPA-NFV, with some atypical features (semantic pharaphasias and the early severe impairment of syntactic comprehension, as well as the cortical atrophy of temporal lobes).
